# Determining the biomechanics of touch sensation in *C. elegans*

**DOI:** 10.1038/s41598-017-12190-0

**Published:** 2017-09-26

**Authors:** Muna Elmi, Vijay M. Pawar, Michael Shaw, David Wong, Haoyun Zhan, Mandayam A. Srinivasan

**Affiliations:** 1 UCL TouchLab, Department of Computer Science, University College London, London, WC1E 6BT UK; 2 National Physical Laboratory, Hampton Road, Teddington, Middlesex TW11 0LW UK; 3 MIT TouchLab, Massachusetts Institute of Technology, Department of Mechanical Engineering and the Research Laboratory of Electronics, Cambridge, MA 02139 USA

## Abstract

The sense of touch is a fundamental mechanism that nearly all organisms use to interact with their surroundings. However, the process of mechanotransduction whereby a mechanical stimulus gives rise to a neuronal response is not well understood. In this paper we present an investigation of the biomechanics of touch using the model organism *C. elegans*. By developing a custom micromanipulation and force sensing system around a high resolution optical microscope, we measured the spatial deformation of the organism’s cuticle and force response to controlled uniaxial indentations. We combined these experimental results with anatomical data to create a multilayer computational biomechanical model of the organism and accurately derive its material properties such as the elastic modulus and poisson’s ratio. We demonstrate the utility of this model by combining it with previously published electrophysiological data to provide quantitative insights into different biomechanical states for mechanotransduction, including the first estimate of the sensitivity of an individual mechanoreceptor to an applied stimulus (parameterised as strain energy density). We also interpret empirical behavioural data to estimate the minimum number of mechanoreceptors which must be activated to elicit a behavioural response.

## Introduction

**Figure 1:**
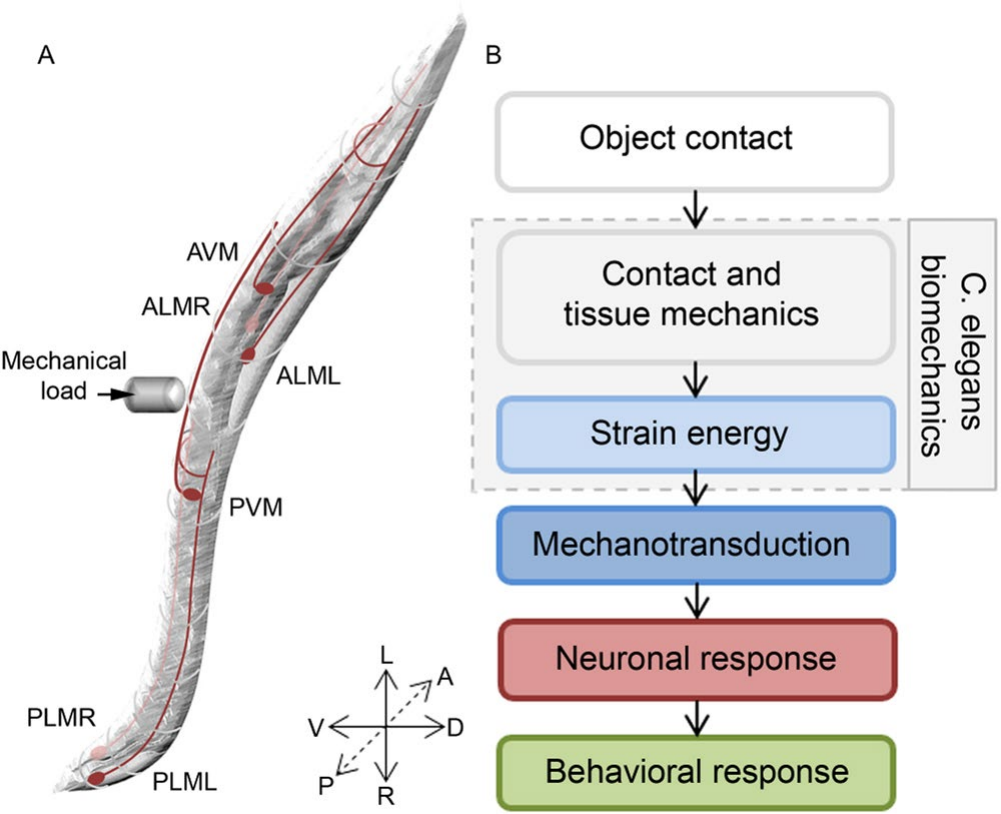
The process of touch sensation. (**A**) Illustration of a *C. elegans* highlighted with its six gentle touch receptor neurons. (**B**) Block diagram describing the different processes related to touch sensation.

The ability to sense touch is a fundamental perceptual mechanism that helps us explore and manipulate our environment. Whilst these physical interactions are ubiquitous to our everyday actions, relatively little is known about the neurophysiological processes used to interpret mechanical phenomena occurring upon contact. As shown in Fig. 1B, touch sensation can be considered as a chain of events initiated by the spatiotemporal distribution of mechanical loads applied to the skin, which result in stresses that deform the skin’s surface, sub-layers and underlying mechanoreceptors^1^. In response to the transmitted mechanical energy, the mechanoreceptors activate a neuronal signaling network that encodes the physical contact as information which we perceive as ‘touch’^2–5^. The details of this process thus depend upon the relationship between physical contact, interface forces, spatial deformation and activation of mechanosensitive substructures. By understanding these mechanical states and biomechanical properties, in addition to their subsequent neuronal and behavioural effects, we can gain new insights into the sense of touch.

With its simple morphology and well defined nervous system, *Caenorhabditis elegans* (*C. elegans*) is a powerful model for understanding the fundamentals of touch sensation^6^. *C. elegans* has six identified gentle touch receptor neurons (TRNs) embedded in the hypodermis that run longitudinally along the body (Fig. 1A). These TRNs can be activated by stroking the cuticle with a fine hair, resulting in predictable avoidance behavioural responses^7–9^. The deformation due to physical contact activates specialized mechanotransduction (MeT) channels distributed along the TRN neurite^10,11^. However, despite recent studies in which more quantitative experimental methods have been applied^10,12–14^, much remains unknown about the fundamentals of touch sensation in *C. elegans* such as the pathway of force transmission to the MeT channel and how the channel is gated.

We can combine data about the anatomy of *C. elegans* and the mechanical properties of its constituent materials in a mechanistic model^4,5^, providing a platform to verify and generate hypotheses at a variety of levels ranging from mechanotransduction to neural activation and behavior. Atomic Force Microscopy (AFM) and micro-electromechanical systems (MEMs) based force sensing systems provide the resolution needed to measure the relationship between force and displacements of the animal’s body under load^15,16^. However, such systems typically occlude the contact interface during indentation, preventing the determination of the displacement distribution over the worm surface, which is critical to understand and quantify the spatial distribution of the stress and strain at the mechanotransduction sites within the organism.

In common with many other biological systems, *C. elegans* consists of materials with complex mechanical properties, including inhomogeneity, anisotropy and viscoelasticity^15,17^. Many studies which have sought to explain the measured force-displacement response of the organism^12,15^ or its locomotion characteristics^17,18^ have approximated *C. elegans* as a thin homogeneous cylindrical shell with internal hydrostatic pressure. Using highly idealized analytical models of *C. elegans* body mechanics, reported values of the elastic modulus (*E*) of the organism range over five orders of magnitude between 4 kPa to 400 MPa^12,13,15,17–20^. Beyond these large variations, at present, very little is known about other mechanical properties such as Poisson’s ratio (*v*) and how properties vary between different layers of the organism. To overcome some of these limitations, we have developed computational model to realistically reflect the geometry and material properties of *C. elegans*. Using this approach, we were able to accurately simulate a range of the applied physical stimuli and real world boundary conditions.

In order to validate the model we developed a custom high-precision microindentation system, comprising of a micromanipulation platform and MEMs based force probe integrated with a high resolution optical microscope. This enabled us to apply a range of controlled mechanical stimuli, whilst simultaneously capturing applied load and resulting spatial deformation of the worm body. By matching the predictions of the model to the empirical results we were able to fine-tune the material properties of a two-layer finite element model (FEM) allowing us to quantify the mechanical state throughout the organism. Following previous studies in primates^4,5,21^, we consider strain energy density (SED) as a candidate for being the primary stimulus for mechanotransduction. By using the model to interpret electrophysiological and behavioural data from previous studies^10,13^, we provide the first estimate of the sensitivity of individual TRNs to SED and also estimate the minimum number of mechanoreceptors which need to be activated for the organism to elicit a measureable behavioural response.

## Results

### Measurement of spatial deformation under controlled mechanical stimuli

**Figure 2:**
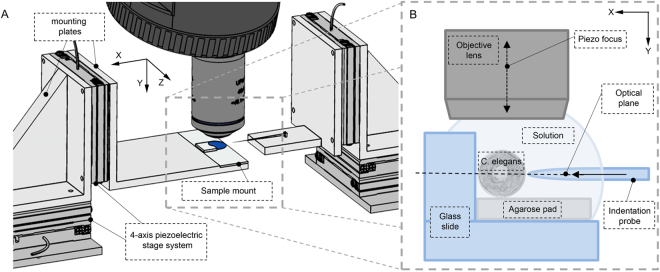
Experimental set up. (**A**) Illustration of the mechanical and optical hardware components. (**B**) Diagram of the sample preparation and probe positioning.

To measure the mechanical properties of *C. elegans*, we developed a high-precision micro-indentation system to allow uniaxial indentation of the worm using a MEMs based microforce sensing probe with a tip 0.7 μm in diameter (Fig. 2). We used this system to capture force-displacement and spatial deformation data for a series of anesthetized worms, using 2,3-butanedione monoxime (BDM). BDM causes flaccid paralysis by relaxing the muscles. The worms were mounted on agarose pads for stability and to provide a rigid surface against which to indent the worms and were glued to the edge of a coverslip using a biocompatible adhesive (Fig. 2B). The glue was only applied minimally between the glass wall and the worm, on the side opposite to the point of indentation. To establish that this preparation procedure did not cause any significant damage to the organism, specimens were observed over a period of several hours following anesthesia and gluing. As for BDM treated worms recovering from paralysis on agar pads, when glued worms were allowed to recover from the anesthetic we observed pharyngeal pumping and head rearing indicating normal, healthy functioning of the organism. Occasionally, depending on the spatial distribution of glue and level of adhesion of the worm to the coverslip, worms were also able to move other parts of their body in a manner we could expect for an untreated, unglued organism.

**Figure 3:**
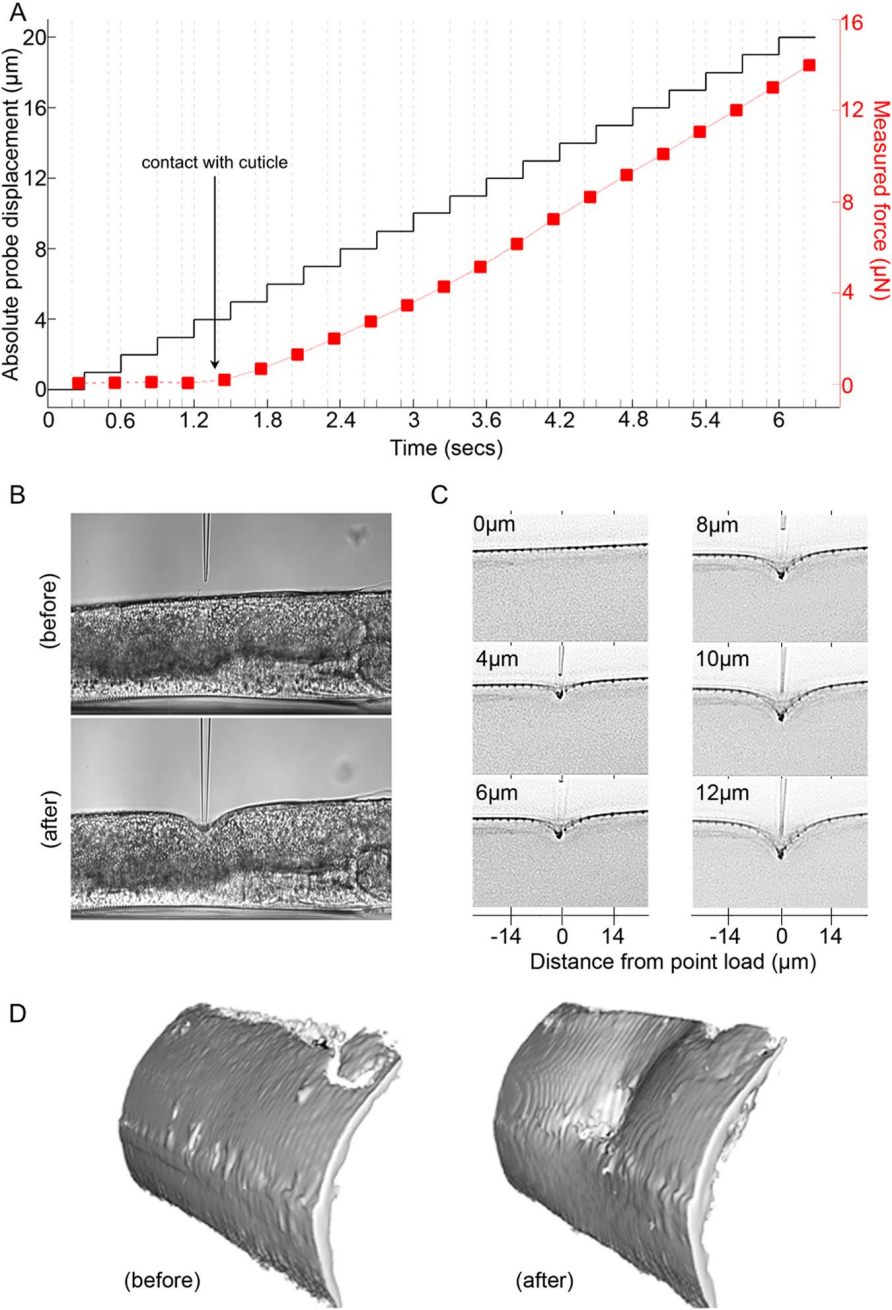
Indentation of *C. elegans*. (**A**) Measurement of displacement and applied force as a function of time for an adult worm indented around its mid-body (**B**) Typical brightfield images captured at 0 µm (before) and at 12 µm (after) indentation. (**C**) Deconvolved fluorescence images showing deformation of the DiI labelled cuticle at different indentation depths (displayed as inverted grayscale). (**D**) 3D isosurface (rendered from deconvolved fluorescence images) showing *C. elegans* cuticle before (left) and after (right) indentation to a depth of 12 µm (right) illustrating the extent and topography of the resulting deformation.

The micromanipulation system was programmed to execute an automated indentation sequence with the applied indention force and focal series of images (z-stack) captured at each indentation depth. By synchronizing the acquisition of optical images and sampling of the output from the force sensor we were able to simultaneously measure the spatial deformation of the cuticle and the applied load (Fig. 3A). We allowed for a delay of 300 ms between each indentation step to allow for viscous relaxation, ensuring measurements under steady state conditions. Figure 3B shows typical cuticle deformation profiles before and after indentation under brightfield illumination. To improve image contrast, and enable effective segmentation of the cuticle using computer vision algorithms, we labeled the cuticle with the fluorescent lipophilic dye (1,1′-diotadecyl-3,3,3′,3′-tetramethylindocarbocyanine perchlorate, DiI). Performing 3D deconvolution on the focal stack of epifluorescene images acquired for each indentation depth resulted in a set of image slices from which the transverse deformation profile could be readily extracted. Shown in Fig. 3C, are representative deconvolved images corresponding to the plane containing the cuticle, spatial deformation transverse to the load, and probe contact point at different depths of indentation. We observed that the transverse spatial deformation profiles were relatively symmetrical about the point of indentation. The 3D isosurface images shown in Fig. 3D indicate that the imaging system was also sensitive enough to capture a degree of anisotropy in the deformation, arising in part due to the cylindrical geometry of *C. elegans*.

**Figure 4:**
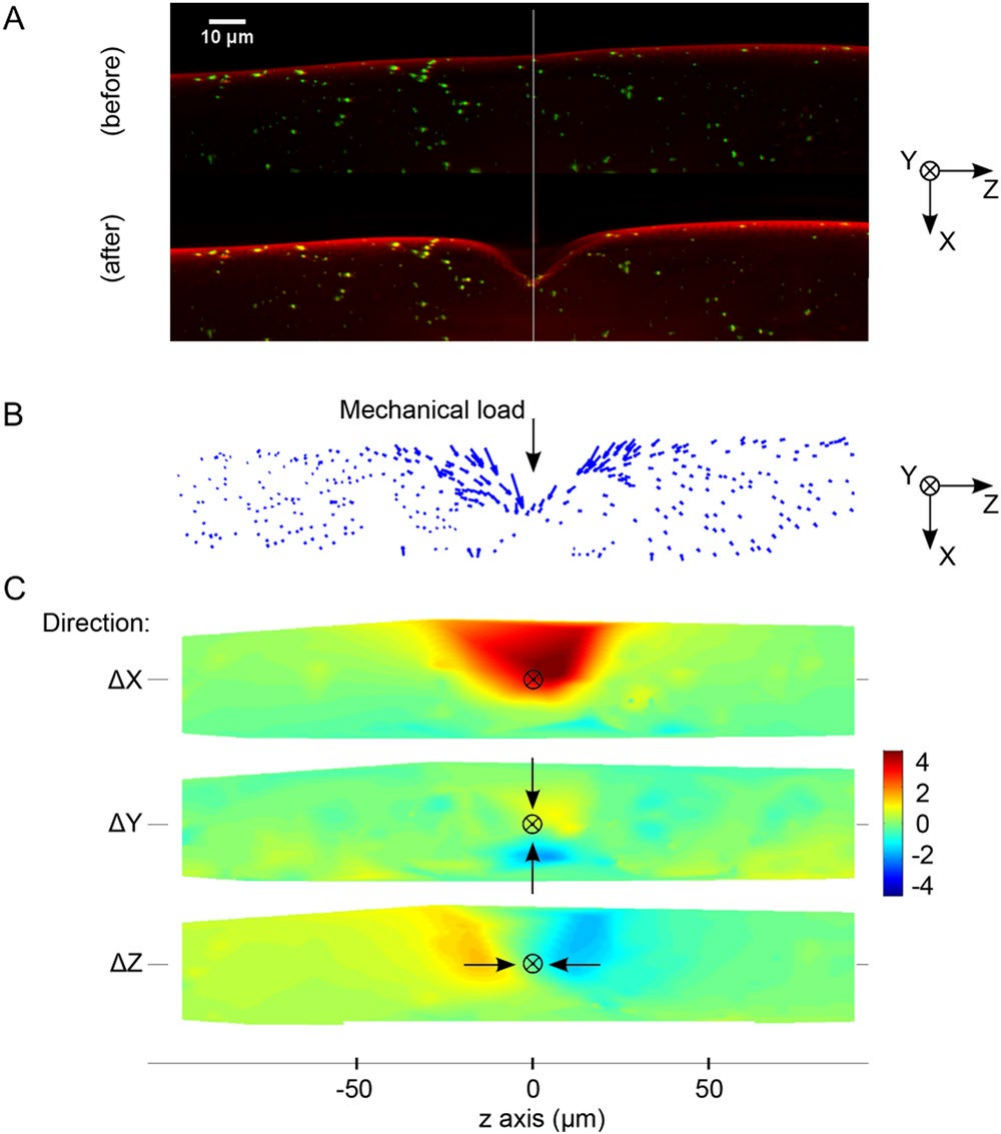
Spatial deformation of *C. elegans* cuticle. (**A**) Fluorescence images showing cuticle with DiI (red) staining and fluorescent microspheres passively absorbed (green), before (top) and after (below) indentation. (**B**) Microsphere trajectories and the spatial position of the individual microspheres at 0 to 4 µm indentation of the worm body depicted as a vector trajectory and (**C**) as a displacement heat map. The heat map shows the magnitude of the cuticle displacement in directions parallel (x) and perpendicular (y and z) to the direction of indentation. The point at which the load is applied to the cuticle is shown by the black crosshair. Arrows represents direction of movement of the beads.

**Figure 5:**
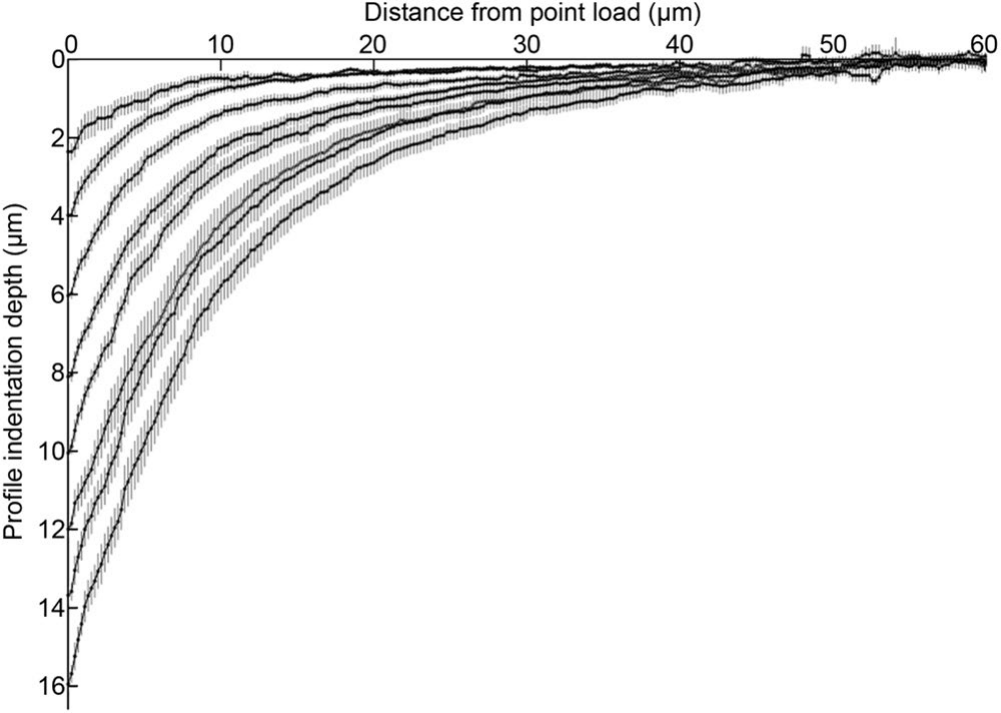
Graph showing the experimentally extracted mean steady state surface deformation profiles of *C. elegans* body (n = 14), after microprobe indentation depth range of 2 to 16 µm. Error bars show + /- s.e.m. Point of load occurring at 0 µm on horizontal axis.

To further investigate the deformation of the worm body under load, we used fluorescent microspheres adsorbed to the outside of the cuticle. Shown in Fig. 4A, the microspheres provided clear markers for feature tracking allowing measurement of displacement trajectories at different locations on the cuticle. Figure 4B shows typical microsphere displacement vectors for an indention depth of 4 µm. Heat map representations in Fig. 4C show the displacements along the indentation axis (X), parallel to the long axis of the animal (Z), and the axial plane (Y) indicating that the cuticle spatial deformation is restricted to a small region (approx. 30 µm) around the point of indentation with no substantial global movement such as bending, rotation or translation of the animal. As expected, we also observed the cuticle deformation to be greatest along the indentation direction X. Displacement along the Z axis were greater than those along the Y axis, consistent with cylinder deformation under transverse point indentation (Fig. 4C). Some asymmetry can be observed on the displacement along the different axes. We attribute this to the fact that *C. elegans* does not in practice have an ideal cylindrical body shape and is compromised of material which are not structurally homogeneous or isotropic. Consequently some asymmetry in the observed deformation is to be expected. Figure 5 shows the cuticle profiles for a range of indentation depths. We observed that indentations of up to 16 µm were still within the elastic regime as the outer surface of the animal fully recovered after indentation.

### Development of a biomechanical model for *C. elegans*

**Figure 6:**
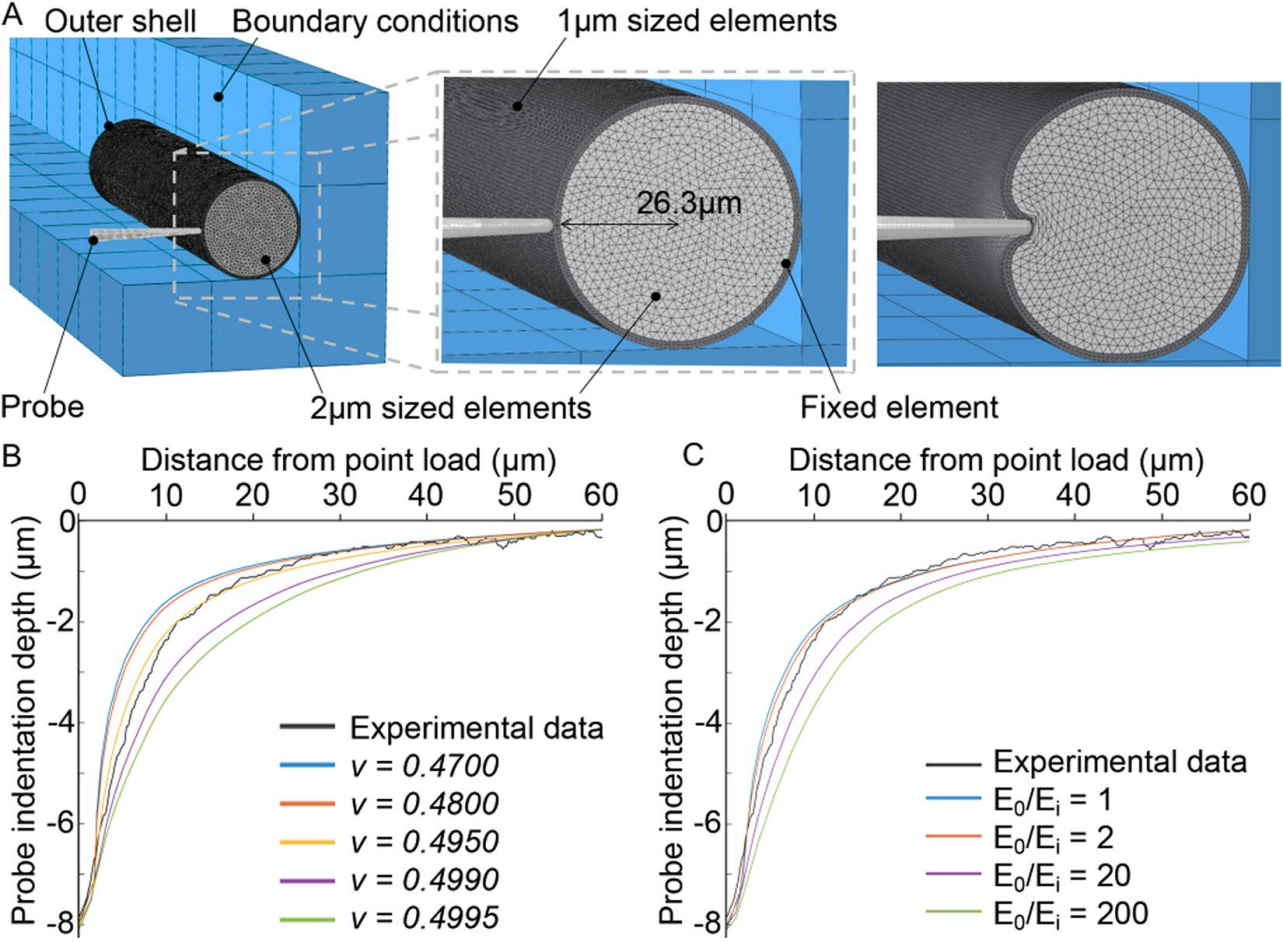
(**A**) A cross-section of the 3D finite element model of *C*. *elegans*, depicting a two- layered structure with an outer layer composed of 1 µm element size, and inner core with 2 µm elements, simulating the experimental conditions. (Far right) Example simulation of depicting spatial deformations of the organism under a point load. (**B**) Comparing computer generated steady state deformation profiles based on different *v* values for *E* ratio = 2 and (**C**) different *E* ratios at 8 µm probe indentations and *v* = 0.495 to determine the material properties of the model which give the best agreement with experimental data.

For modelling purposes, as shown in Fig. 6, we defined the geometry of the simulated organism as a pressurized cylinder composed of two layers with dimensions based on the reported anatomy^22^: 1) The outer layer with a total thickness of 1.7 µm, represents the cuticle (0.6 µm), the hypodermis (0.1 µm) and the body wall muscles (1.0 µm)^15,23^. 2) The inner layer was assigned a radius of 26.3 µm giving a total body diameter of 56 μm, equal to the mean diameter of all the animals used in our study. To increase the spatial resolution and improve the accuracy of the model close to the TRNs, we set the element size for the outer layer to be half the size (1 µm) of that for the inner layer (2 µm).

We followed the principle of parsimony to minimize the number of material parameters required for the model to accurately match the empirical data. Due to limitations in the current literature describing the material properties for *C. elegans*, we constrained the materials comprising the inner and outer layers to be linear elastic, homogeneous and isotropic. By using these simplifications, the inner and outer layers are each described by two parameters: Poisson’s ratio (*v*), a measure of compressibility or volume change of the material and Young’s modulus of elasticity (*E*). As biological tissues are mostly composed of water, we further assumed the inner and outer layers to have the same volumetric incompressibility, further reducing the complexity of the model. Other factors such as the effect of the internal pressure necessary for the worm to maintain its body shape was also considered^23^. For simplicity, the resultant simulation outputs using our two-layer model did not account for any contact inertia or material damping effects as the measured spatial deformation profiles were captured only when the cuticle had reached a steady state.

### Validation of the biomechanical model

As discussed by Srinivasan and Dandekar^4^ and Dandekar *et al*.^5^, when both the loading stimulus applied to a multilayered elastic object and its boundary conditions are specified in terms of displacements, the resulting spatial deformation depends only on the ratio *E*_*o*_/*E*_*i*_ (stiffness of the outer layer *E*_*o*_/stiffness of the inner layer *E*_*i*_) of the layers and not the absolute values of *E*. By comparing the simulated spatial deformation profiles of the cuticle to the empirical data, we optimised the parameters of the model through tests investigating the differences due to: 1) the *v* value, 2) the *E*_*o*_ */E*_*i*_ (Fig. 6B,C), 3) and internal pressure (Figure S1). In all cases, the best fit was determined by calculating and selecting the smallest root mean square error between the simulated and empirical data sets (Table S1) over a range of indentation depths. Since the experimental data obtained in this work is from BDM paralysed worms, our results describe the *C. elegans* body mechanics when in the relaxed muscle state.

Similar to other nematodes, such as *Ascaris*, which has the internal pressure of up to 30 kPa^24^, *C. elegans* maintains its body shape by internal hydrostatic pressure^23^. To model the behavior of the layers and to investigate the effect of internal pressure, we experimented with different values of internal pressure ranging from 0.4–40 kPa. As shown (Figure S1), we found that the internal pressure did not have any effect on the spatial deformation. This suggests that hydrostatic pressure has little effect on mechanotransduction of external stimuli, since this depends only on the change in stress/strain state at the transduction site (MeT channel). Moreover, there was only a small effect when the pressure is increased from 4 kPa to 40 kPa (Figure S2). Subsequently it was excluded when tuning the model parameters. Interestingly, our model prediction that the internal pressure has little effect on mechanotransduction agrees with the previous finding that when internal pressure in *C. elegans* is reduced, the TRNs still remain functional and responsive to mechanical stimuli^10^.

We found that a ratio of elastic moduli (*E*_*o*_/*E*_*i*_) of less than one yielded model predictions which resulted in a poor fit with the empirical data (Figure S3). In this case the resistance exerted by the stiffer inner layer caused compression of the outer layer that was inconsistent with the measured spatial deformations along the length of the animal. Based on this result, we constrained our optimization to *E* ratios higher than 1. Further, as shown in Fig. 6C, preliminary simulation results suggested that *E*_*o*_/*E*_*i*_ was smaller than 20.

**Figure 7:**
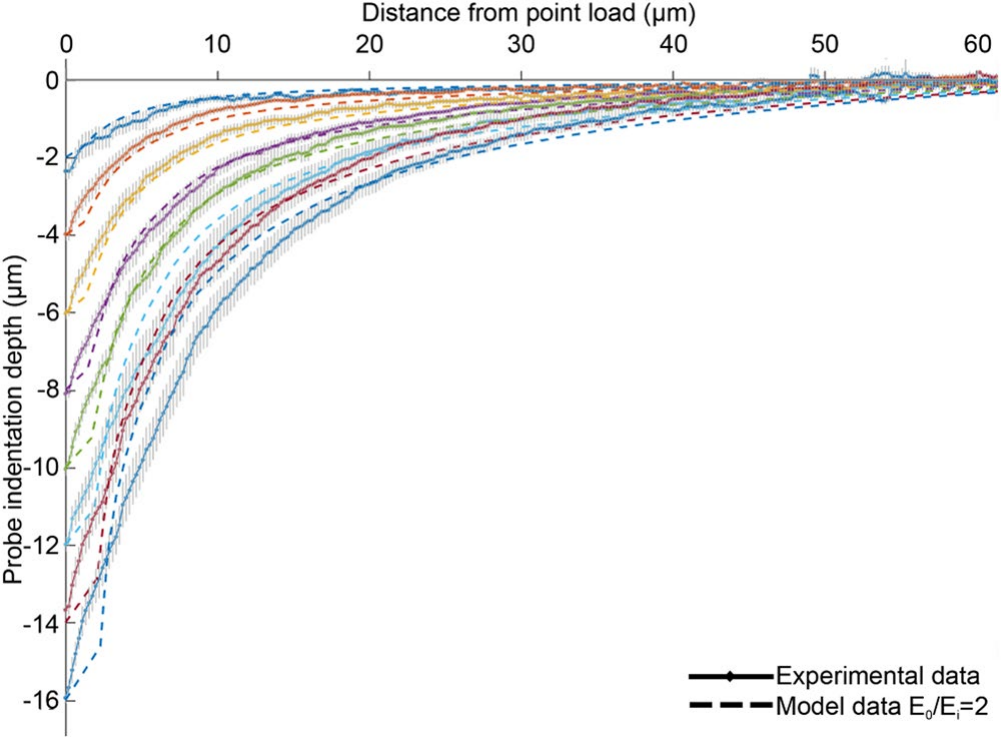
Comparison of surface deformation profiles between mean experimental data (solid lines) and numerical prediction (dashed lines) using *E*_*o*_ = 140 kPa, *E*_*i*_ = 70 kPa and *v* = 0.495. Each color represents and indentation depth.

We found that simulated spatial deformation profiles were sensitive to both *v* and *E*_*o*_/*E*_*i*_. For 0.3 < *v* < *0.4*, it was not possible to match empirical deformation profiles. Using this reduced parameter space, 1 < *E*_*o*_/*E*_*i*_ < 20 and *v  < 0.4*, we found optimal values for *v* and *E*_*o*_/*E*_i_ to be 0.495 and 2 respectively for indentations up to and including 10 µm (Fig. 7). For the larger indentations in the range 12–16 µm, we found that *E*_*o*_/*E*_*i*_ increased up to between 5 and 8 while keeping *v* = 0.495 resulted in simulated spatial profiles that best matched the experimental results (Figure S4). However, for these larger indentations we observed only subtle differences in the predicted profiles by the model as *E*_*o*_/*E*_*i*_ was increased. Consistent with observation by Dandekar *et al*.^5^, fixing *E*_*o*_/*E*_*i*_ while changing the absolute values of *E*_*o*_ and *E*_*i*_ did not have an effect on the shape of the simulated spatial deformation profiles.

**Figure 8:**
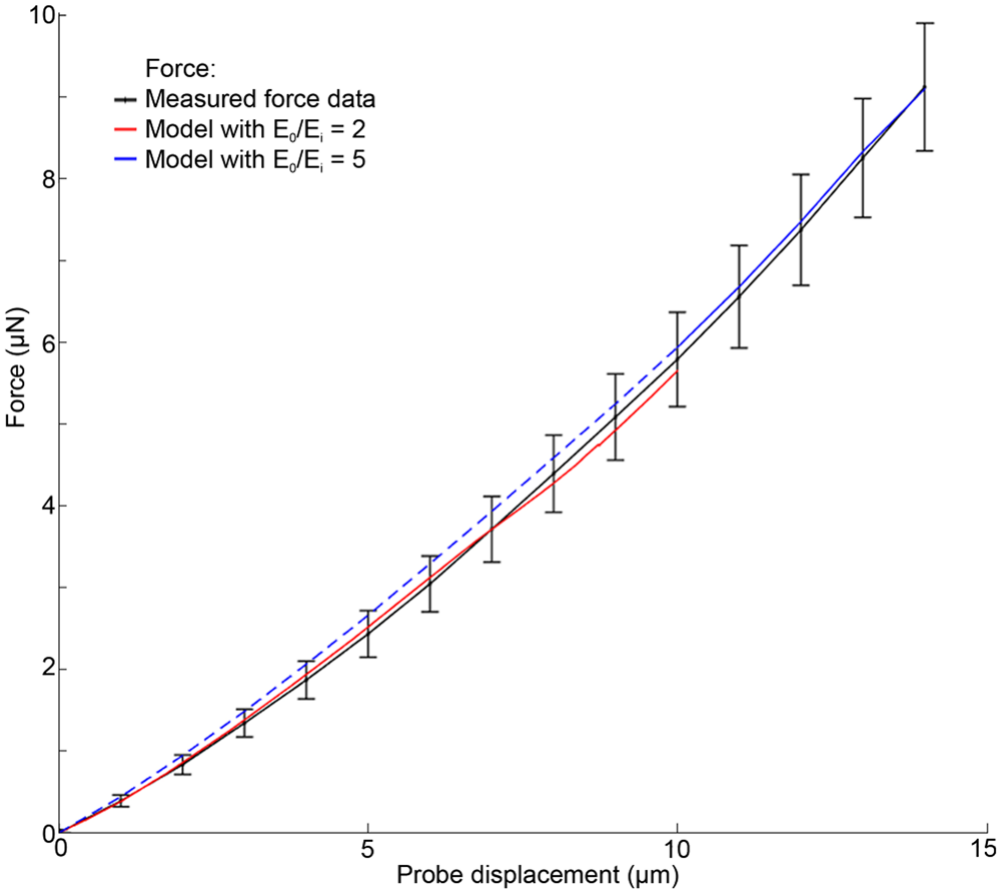
Average force-displacement measurement (black line) using force-sensing microprobes, indenting the animal in a region close to the vulva either posteriorly or anteriorly (n = 9 animals). Computer simulation using an absolute *E*_*o*_ and *E*_*i*_ value of 140/70 kPa (red line) and 300/60 kPa (blue line) respectively to find a matching force-displacement curve to the mean of experimentally obtained measurements. Blue dashed line is 0–10 µm and blue solid line is 10–14 µm. Values are the averaged data ± s.e.m.

To determine the absolute values of *E*_*o*_ and *E*_*i*_, we performed a series of force-displacement measurements. As shown in Fig. 8, our measurements indicate that as the indentation depth increases, the reaction force increases with an observable nonlinearity. Our model simulations indicate that this nonlinearity is initially due to the contact mechanics (change in contact area between the probe and cuticle). But as indentation depth increases beyond ~1 μm, both geometric nonlinearity due to large deformation (taken into account by the model) and material nonlinearity (approximated as piecewise linear material by the different *E*_*o*_/*E*_*i*_ ratios for small and large indentations) are indicated in the force-displacement data. Across all measured indentation conditions we calculated an average linear worm stiffness of 0.62 + /−0.17 N/m which agrees well with previously reported findings^12,15^. To investigate whether the worm stiffness changed over the duration of an experiment, we measured the force-displacement characteristic of several worms immediately after mounting (t_0_) and 30 minutes later (t_1_). Figure S5 shows the mean normalized stiffness at these two points indicating no substantial change in stiffness over time.

To further validate the model, we performed a second set of simulations to test the goodness of fit between the measured force-displacement response of the organism and that predicted by the model (Fig. 8). The magnitudes of *E*_*o*_ and *E*_*i*_ were determined by iteratively refining their values while keeping the ratio *E*_*o*_/*E*_*i*_ constant until the best fit between the simulated and empirical force-displacement curves was achieved. The experimentally obtained force-displacement data has an average + /−11% standard error of the mean force, and in our model the indentation force is proportional to E. Therefore, our results indicate that on average, the error in *E*_*o*_ and *E*_*i*_ estimates are approximately within + /−11%.

Since the analysis involving spatial deformation profiles suggested different values of *E*_*o*_/*E*_*i*_ for small (≤10 μm) and large (>10 μm) indentation we obtained the *E*_*o*_ and *E*_*i*_ values for each of these indentation ranges separately. For small indentation depths (*E*_*o*_ */E*_*i*_ = 2), we found the optimal match between the model and empirical force-displacement data suggested *E*_*o*_ = 140 kPa and *E*_*i*_ = 70 kPa. For large indentations we found optimal values of *E*_*o*_ = 300 kPa and *E*_*i*_ = 60 kPa. It is fairly common in non-linear materials that as the deformation increases the material becomes stiffer. Our results suggest that the increase in body stiffness with indentation depth is mainly due to the material non-linearity of the outer layer, which in turn, accounts for the larger value of *E*_*o*_/*E*_*i*_ at higher indentations. Our model with different *E*_*o*_/*E*_*i*_ values for small and large indentations, represents a piecewise linear approximation of the material nonlinearity observed in the experimentally measured data.

### Model Predictions

**Figure 9:**
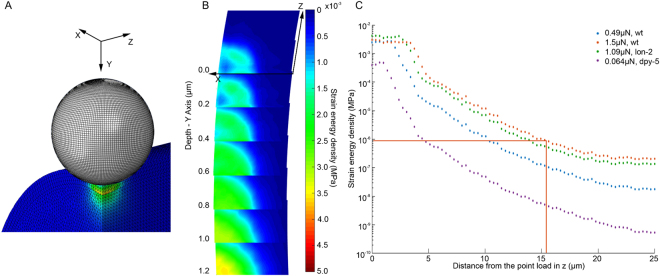
(**A**) Finite element model of *C*. *elegans* body deformed by a 10 µm diameter sphere with a 0.49 µN stimulus. (**B**) A heat map distribution of SED at the receptor location (ie. 0.6 µm below surface) after 0.49 µN mechanical load. The y axis is the location of the probe as it was indented, while the z axis is the longitudinal and x axis transverse direction of the worm body. Each layer is a slice of 0.2 µm in depth (y axis) and the plane is 4 µm × 4 µm (x and z axis respectively). (**C**) At the depth of 0.5–0.7 µm where the neurites of the touch neurons reside the SED distribution along the length of the worm (z direction) within a ROI of 200 nm after force stimulation of 0.064 µN, 0.49 µN, or 1.09 µN with a 10 µm diameter probe or 1.5 µN with a 15 µm diameter probe (red line) respectively.

We were able to apply our model predict and visualize the 3D spatial distribution of different mechanical states of the organism in response to any defined force or displacement stimulus. Further, by combining the model data with electrophysiological and behavioural data^10,13^, we were able to relate this information to the mechanical state of the organism required to activate individual MeT channel sites. Proposed by Srinivasan and Dandekar^4^, and supported by other studies^21^, we chose Strain Energy Density (SED) as a candidate for the primary mechanical trigger that activates the MeT channel sites. Figure 9 shows the SED distribution in the organism when it is indented with a 10 μm sphere with a force of 0.49 μN. An implication of this model is that the peak SED occurs not at the point of contact with the indentation probe, but deeper within the cuticle closer to the actual location of the MeT channels. This shift arises due to the difference in *E* of the outer and inner layers and is not observed in homogenous (non-layered) models (see Figure S6). This would have to be validated in future research.

We can interrogate our model to test various hypotheses about mechanosensation in *C. elegans*. There have been several studies to establish the mechanical sensitivity of *C. elegans*. O’Hagan *et al*.^10^ used patch clamp electrophysiology to measure the invoked mechanosensory current (MRC) following a defined force load applied to the worm cuticle. Using a spherical indentor they found that forces of 1–2 µN saturated the MRC response, suggesting that all the MeT channels available to be opened are activated at this force. At ion current saturation, O’Hagan *et al*.^10^ predicted a minimum of 14 MeT channels are open. Assuming an interchannel spacing of 2.4 µm^25^, and that indentation occurs directly above the neurite where the channels sit, this suggests the most distant open channel is 15.6 µm from the point of indentation. Figure 9C shows how SED varies along the neurite (perpendicular to the plane of indentation and parallel to the long axis of the worm). At a distance of 15.6 µm from the indentation point the SED is 0.84 Pa (see red line Fig. 9C), which can be considered as the SED threshold required for activation of a single MeT channel.

Based on this SED threshold we can further use our model to estimate the number of channels required to elicit a behavioural response. In a different experiment, Petzold *et al*.^13^ reported the indentation force required for a behavioural response in wild type worms to be 0.49 µN using a 10 µm spherical indentor^13^. Using our two-layered model adjusted to their experimental conditions and based on their force-displacement data, we estimated the *E*_*o*_/*E*_*i*_ to be 200/100 kPa, *E* values very similar to our findings. If the SED necessary to open a MeT channel is 0.84 Pa, our model predicts that an applied force of 0.49 µN would activate 8 MeT channels (Fig. 9C); where, as previously, we assume that the TRN sits directly underneath the probe contact point.

We also investigated how the number of channels needed to activate a behavioural response varies with cuticle stiffness (*E*_*o*_) and MeT channel spacing. Petzold *et al*.^13^ report that for mutant worms *dpy-5* with lower body stiffness (0.39 N/m), an estimated force of only 0.064 µN was sufficient to elicit a behavioural response. When we simulated this experiment, the SED dropped below the channel activation threshold of 0.84 Pa at a distance of 4.77 µm from the point of load. As *dpy-5* has a similar MeT channel space to wild-type^13^, this suggests that only 4 MeTs were activated. While in another mutant *lon-2*, for which MeT channel spacing increased by approximately 14%, a greater force of 1.09 µN is required to elicit a behavioural response^13^. In this case our model predicted that 10 channels were activated for that response (Fig. 9C). Interpreting these experiments using our biomechanical model thus suggests that the minimum number of MeT channels required to elicit a behavioural response varies among different mutants. It should be noted however that the disparity in the outcome of the number of channels activated in the different worms may be due to a degree of inaccuracy in the empirical data as some of them are interpolated rather than experimentally measured^10,13^.

## Discussion

To understand both touch sensation and locomotion in *C. elegans* requires knowledge about the biomechanics of the organism. However, touch sensation describes the response to local deformation of the cuticle^13,15^ whereas locomotion involves deformation of the body as a whole^17–19^. Consequently, the means by which the mechanical properties of *C. elegans* are estimated in the literature depend on the research goal. Researchers investigating touch have typically stimulated the organism’s surface with small mechanical probes resulting in local deformation of the cuticle, whereas those investigating locomotion focus on global bending of the organism. In both cases force vs. displacement data is obtained empirically and matched with the predictions of an (typically highly idealized) analytical model to estimate *E* and the materials comprising the animal are assumed to be incompressible (*v* = 0.5). In studies of touch sensation, the worm is typically approximated as a cylindrical shell with a pressurized interior, and the contribution of the inner tissues and organs is ignored^12,13,15^. Models used in locomotion research are generally either cylindrical shells or uniform rods composed of a homogenous material where any differences in the mechanical properties of the cuticle and the inner core are ignored^17–19^.

Such previous studies have resulted in estimates of *E* ranging over five orders of magnitude, from ~4 kPa to 400 MPa. Shell models give rise to higher estimates (because a thin material sheet has to resist the full load), while homogenous models lead to lower estimates. These approaches have been limited by: 1) the use of unrealistic, highly idealized models developed for mathematical expediency which ignores essential features of *C. elegans* anatomy and biomechanics and 2) limited experimental data with which to validate the model. In order to understand the complex mechanical behavior of a biological system such as *C. elegans*, and to realistically represent the loading and boundary conditions, idealized analytical models are inadequate. Furthermore, we have found that force-displacement data alone is insensitive to wide variations in model parameters. In the linear range, a ‘black box’ linear elastic model with any geometry can be made to fit the force-displacement data. Taking into account the empirically determined spatial displacement distribution around the load provides additional information to determine which of these models are valid.

In this paper, we followed the approach developed by Srinivasan and coworkers^1,4,5^ for understanding mechanotransduction in primates. Biomechanical experiments are performed to generate spatial deformation and force displacement data with which to determine the material parameters of the model by matching empirical results with model computations. This approach requires simple experimental loading and boundary conditions that can be mimicked well in the model and following the principle of parsimony; the model should have as few parameters as possible to explain empirical data. By capturing not only force-displacement data, but also the associated spatial deformation, we were able to more fully capture the response of the organism to mechanical stimulation. In particular, we found that deformation data is essential in order to validate the model parameters.

In order to develop a realistic computational model with a minimum number of parameters, we assumed: 1) the organism is a two layered cylinder, thus taking into account the contributions of both the cuticle and the inner core in resisting applied loads; 2) constituent materials are linear elastic for the static loading case, in order to restrict the number of material parameters to *E*_*o*_, *E*_i_ and *v*; 3) the organism is fixed and undergoes no global displacement under indentation (mimicking the mounting conditions used in the experiments). Under these assumptions we found for small indentations (≤10 µm) *E*_*o*_ = 140 kPa and *E*_*i*_ = 70 kPa and for larger indentations (>10 µm) *E*_*o*_ = 300 kPa and *E*_*i*_ = 60 kPa. In other words the increase in body stiffness observed with increased indentation is mainly due to the increase of stiffness for the outer layer.

In the present work we concentrated on forces acting upon the MeT channels, however these findings could also have implications for locomotion as the gentle touch neurons have been shown to be involved in modulating the worms movement^26,27^. Although our experiments were done under steady state conditions, it is to be expected that the organism exhibits viscoelasticity^17^ under non-static conditions. The micromanipulation system used here, offers the possibility of measuring the time dependence of spatial deformation and force-displacement responses^28^. By again matching empirical data to model predictions, it would be possible to determine viscoelastic parameter values to the layers in our model and investigate temporal aspects of MeT channel activation.

## Materials and Methods

### *C. elegans* maintenance and preparation

*C. elegans* wild-type (N2) animals obtained from the Caenorhabditis Genetics Center were used for this study. The animals were maintained using standard normal growth conditions and procedures^29^. For experimental purpose L4 animals were grown at 21 °C until reaching adult stage. To visualize the worm outer surface the young adults were first stained with the fluorescent lipophilic dye DiI (1,1′-dioctadecyl-3,3,3′,3′-tetramethylindocarbocyanine perchlorate) according to reference^30^. For coating the worm surface with beads, the DiI pre-stained worms were incubated for 1 hr in amine-modified fluorescent beads (Invitrogen F8764, with a diameter of 0.2 µm) diluted 1:500 in M9 buffer. Prior to the experiment the worms were immobilized by 40 min treatment with 15 mg/ml BDM (2,3-butanedione monoxime, Sigma-Aldrich). Using cover glass with a 2% agarose pad, the paralyzed worms were then glued on to the edge of a second cover slip using dermabond glue (2-octyl cyanoacrylate, Suturonline.com) and subsequently immersed in M9 buffer.

### Image acquisition and indentation

Images were acquired using an upright widefield fluorescence microscope (BX51WI, Olympus) with a 60x/1.0 water immersion objective lens (LUMPlanFL N, Olympus), dual band GFP/RFP fluorescence filter cube (Semrock) and a scientific CMOS camera (Orca-Flash4.0 v2, Hamamatsu Photonics). Focal series of image were obtained by translating the objective lens using a piezoelectric translation stage (PIFOC, PI) synchronized to the global exposure period of the camera’s rolling shutter. The cuticle of each animal was indented using either a pulled glass capillary with a tip diameter of between 2–4 µm or a microforce sensing probe (FT-S100, FemtoTools) fitted with tungsten tip with a nominal diameter of less than 2 µm. The position of the probe was controlled using a motorized 4-axis stage system (ECS series, Attocube), which allowed precise positioning of the tip within (x, z) and perpendicular (y) to the focal plane of the microscope, as well as adjustment of the in-plane tilt. Animals were mounted on a separate kinematic stage system decoupled from the microscope body and the capillary.

### Image processing and analysis

As a first step acquired DiI and microsphere focal-stacks were cropped to leave only the boundary of the animal. The focal-stacks were then deconvolved using an iterative 3D deconvolution algorithm implemented in ImageJ^31^, with ten iterations and an initial PSF estimate computed assuming Fraunhofer diffraction and nominal system parameters (NA and wavelength). Even for such a relatively large sample we found that this deconvolution procedure resulted in very effective removal of out of focus light, due to the high signal-to-noise ratio and low background level in the images, enabling 3D reconstruction of the gross morphology of the cuticle from the DiI images. Global drifts in the specimen position which occurred between successive z-stacks were corrected using 3D descriptor-based series registration^32^ with the same 3D rigid body geometric transformation applied to the microsphere and DiI image stacks at each capillary indentation depth. The 3D coordinates of individual micropheres on the cuticle were determined from the deconvolved, registered image stacks by center of mass centroiding. The trajectories of individual microspheres were then tracked throughout the indentation series by identifying the same microsphere in successive focal stacks using a constrained nearest neighbor approach.

### Finite element modelling

Due to severe geometric nonlinearities in contact, full implicit finite element solvers were adopted to maintain accuracy of the solution (where Newton-Raphson iterations were applied after each incremental displacement step to enforce equilibrium of the internal structural forces with the externally applied loads^31^). The Jacobian matrix of the system was defined by ensuring the estimated solution was within the convergence radius of the algorithm. The kinetic energy was monitored during simulations and observed to be 0. The displacement analysis also combined the strain energy integral with a minimization process. The potential energy of the systems was written in terms of the nodal displacements and then minimized^32^:

$$[{\rm{K}}]\backslash \{{\rm{Y}}\backslash \}-\backslash \{{\rm{F}}\backslash \}$$

where [K] is the global stiffness matrix; {Y\} is the nodal displacement vector; {F\} is the global force vector.

The changes in structural geometry were taken into account at each displacement step such that the stiffness matrix was updated using the current nodal positions. No inertia or material damping effect was considered in this model. To match our experimental protocol, images were taken for each steady state indentation with a known distance. A friction model was not used since the coefficients for contact between the glass capillary and the cuticle, as well as the contact between the worm and glass wall, were unknown. Instead, boundary conditions were defined to constrain the model and simulate the physical experimental conditions. These constraints ensured that the indentation direction was uniaxial and the element nodes making contact with the capillary tip did not shift or slip during the indentation. The constraints also agreed with the experiments, where the capillary tip makes firm contact with the cuticle surface and did not slip during indentation. Since the stiffness of the glass wall and the glue were much higher than that of the worm, we considered them together as a rigid body. In our model, the effect of the glue, which was used to align and fix the body of *C. elegans* onto the glass wall, was taken into account in the boundary conditions. Since it was assumed that the indentation was symmetrical, only half of the body was simulated with a cylinder length of 500 µm. Further modelling tests showed that a half worm with a length of 250 µm can replace the original length of 500 µm and obtain the same modelling results. Since the touch receptor neurons are situated within the cuticle and we are interested in a quantitative study of the deformation of outer layer, we make the element size of outer layer to be 1 µm and the element size of inner layer to be 2 µm. The inner surface of the outer layer and the outer surface of the inner layer were tied together using the surface-to-surface constraint. This meshing scheme created 553362 elements for the cuticle and 303754 elements for the inner layers. The geometry of the glass capillary was built based on image measurements, where the tip had an average diameter of 0.7 µm. Due to the higher stiffness of the glass capillary, it was also considered as a rigid body. Our testing showed that linear tetrahedral element (4-node tetrahedral C3D4 element type) could model the cylinder under a relatively large local deformation compared to quadratic elements. To keep the triangular elements equilateral, the element size was kept uniform through the length of the worm and no partition or mesh refinement was done on either layer. The solution accuracy was maintained by using a large number of fine elements and convergence tests.

### Model steps

The simulation consists of two main steps: 1) application of a 4 kPa uniform pressure onto the inner surface of the outer layer to simulate hydrostatic pressure. The first step enabled the modeled worm to achieve a steady-state ready for subsequent indention experiments. 2) indentations were all simulated with displacement-controlled boundary conditions. The simulation of the model with nearly 1 million elements and with an indentation depth up to 12 µm took approx. 16 hours using a Xeon workstation with a 6-core, 12 threads 3.2 GHz CPU and 16 GB memory. The memory requirement is relatively high, where ~16 GB memory is required for the simulation.

## Electronic supplementary material

Supplementary Information
