# Supplementary material for: Determining the biomechanics of touch sensation in C. elegans
Source: Sci Rep. 2017 Sep 26;7:12329. doi: 10.1038/s41598-017-12190-0 (PMC5615042; doi:10.1038/s41598-017-12190-0)
Supplement: Supplementary file 1 — Supplementary Information [file 41598_2017_12190_MOESM1_ESM.pdf]

Supporting Information

Determining the biomechanics of touch sensation in *C. elegans*

Muna Elmi, Vijay M. Pawar, Michael Shaw, David Wong[a], Haoyun Zhan, Mandayam A. Srinivasan

A

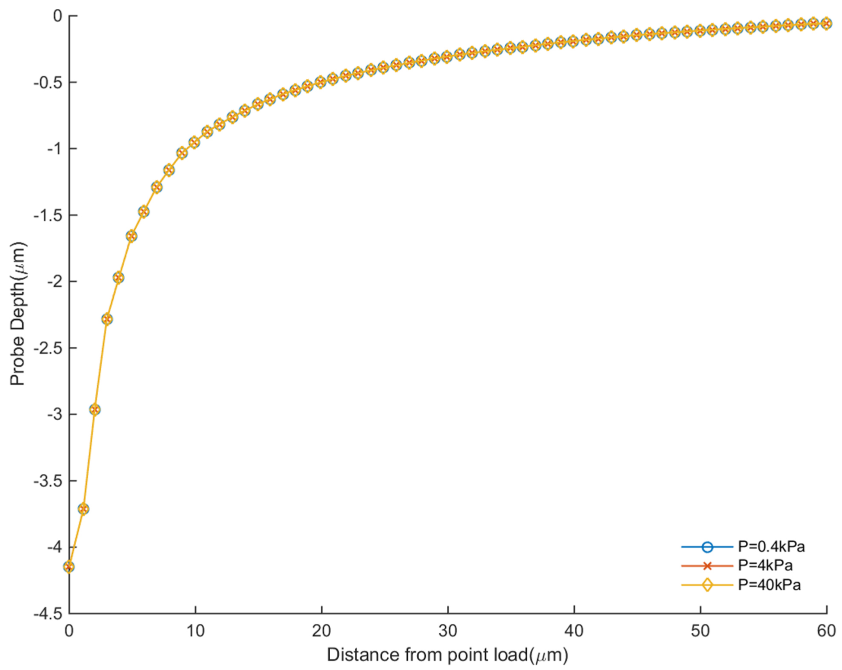

B

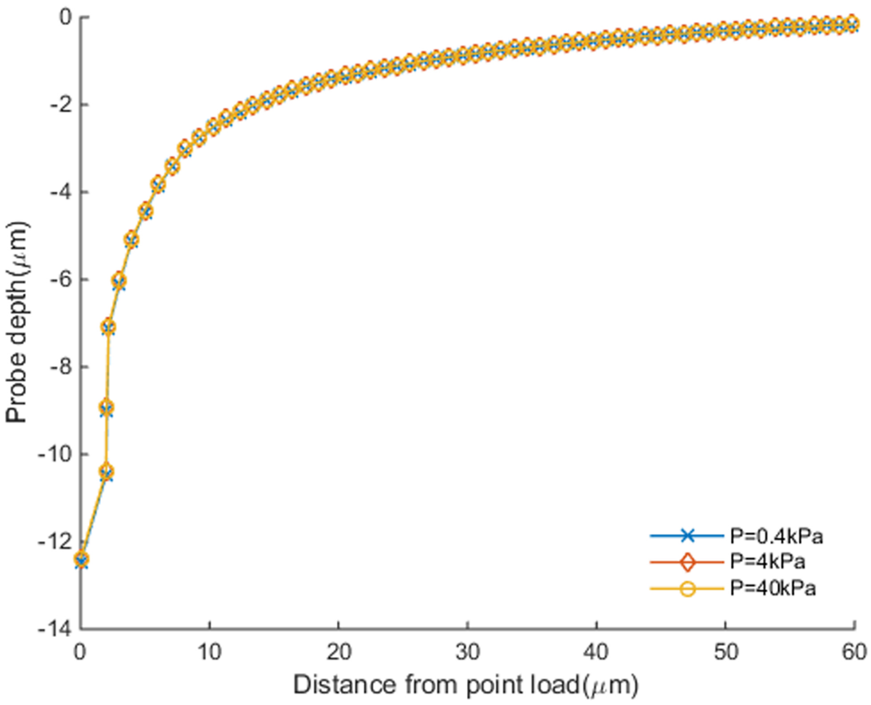

Fig. S1.
A graph showing the computer simulated surface deformation profiles of *C.elegans* body using E_o = 140 kPa, E_i = 70 kPa and v = 0.495 at **(A)** 4 μm, and **(B)** 12 μm indentation, while varying the internal pressure.

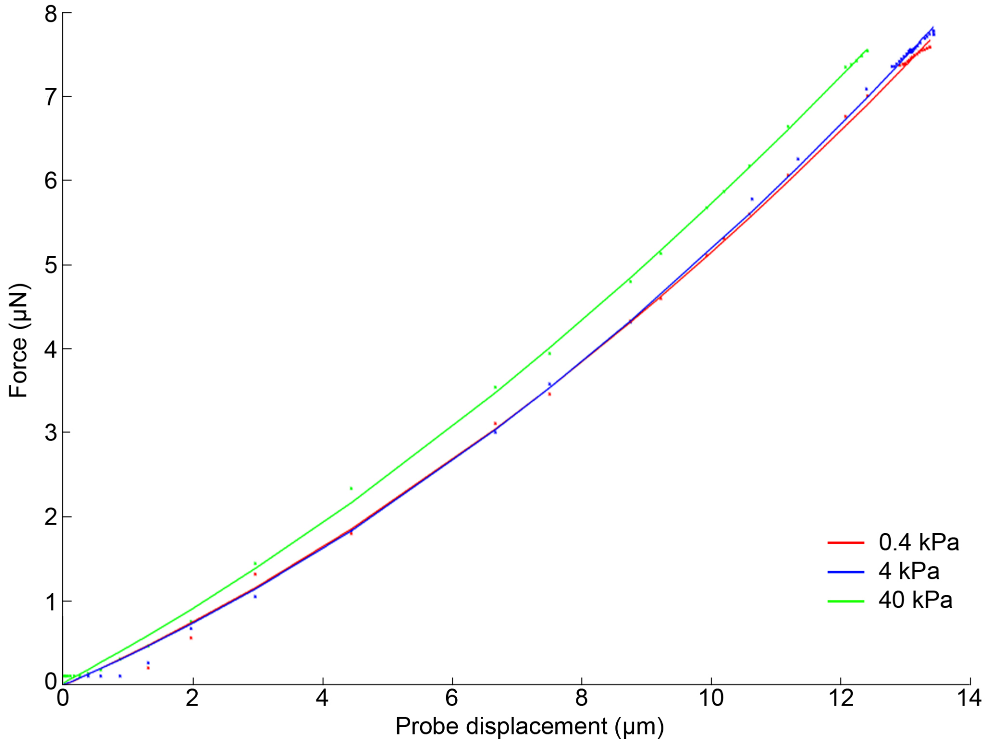

Fig. S2.

Force-displacement data from computer simulated experiments using E_o = 140 kPa, E_i = 70 kPa and v = 0.495 with varying internal pressure between 0.4-40 kPa.

A

B

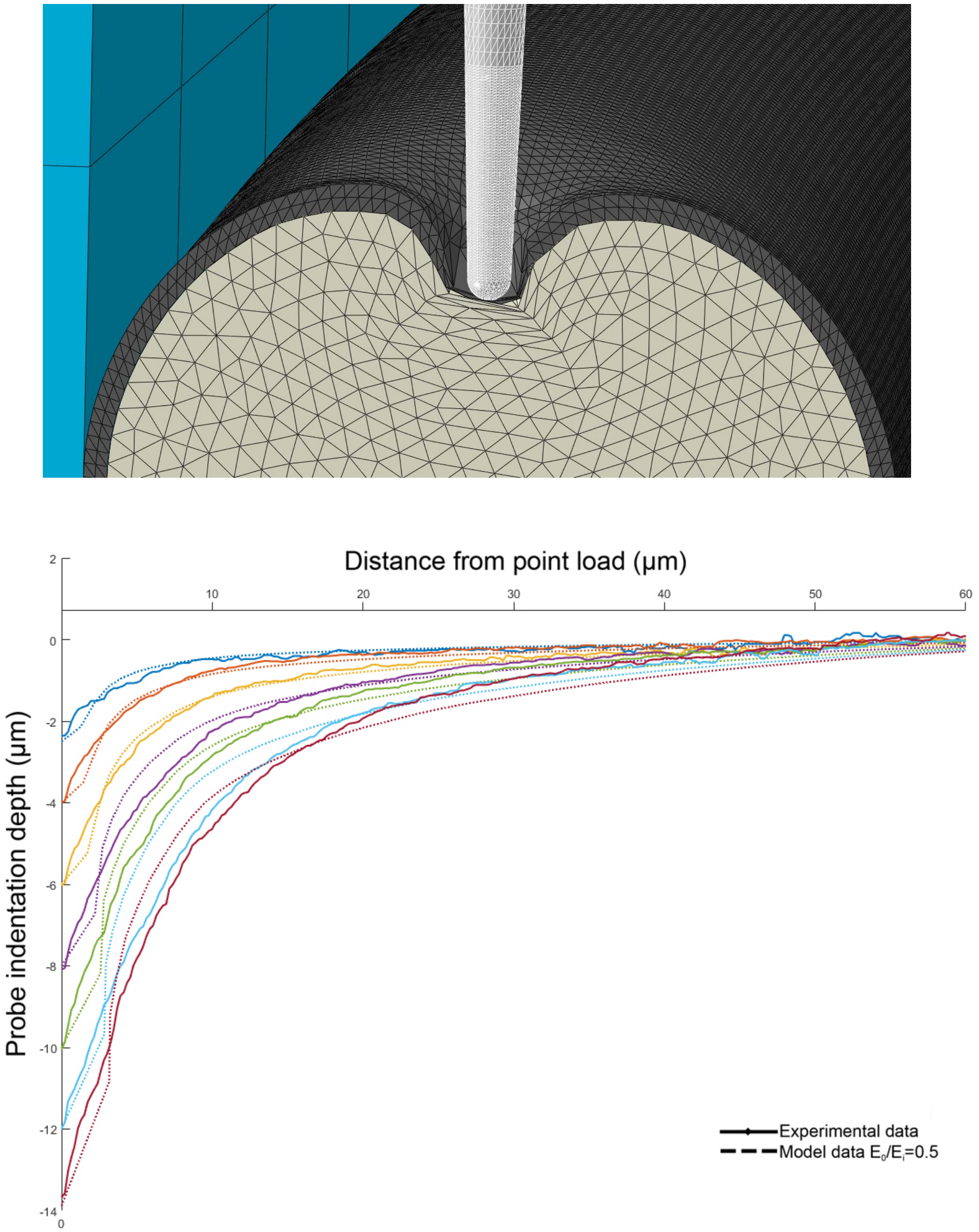

Fig. S3.

A cross-sectional finite element simulation of *C.elegans* depicting a two-layered model with an *E* ratio of 0.5, **(A)** showing compression under the indenting probe and **(B)** the extracted deformation profiles (each color represents an indentation depth).

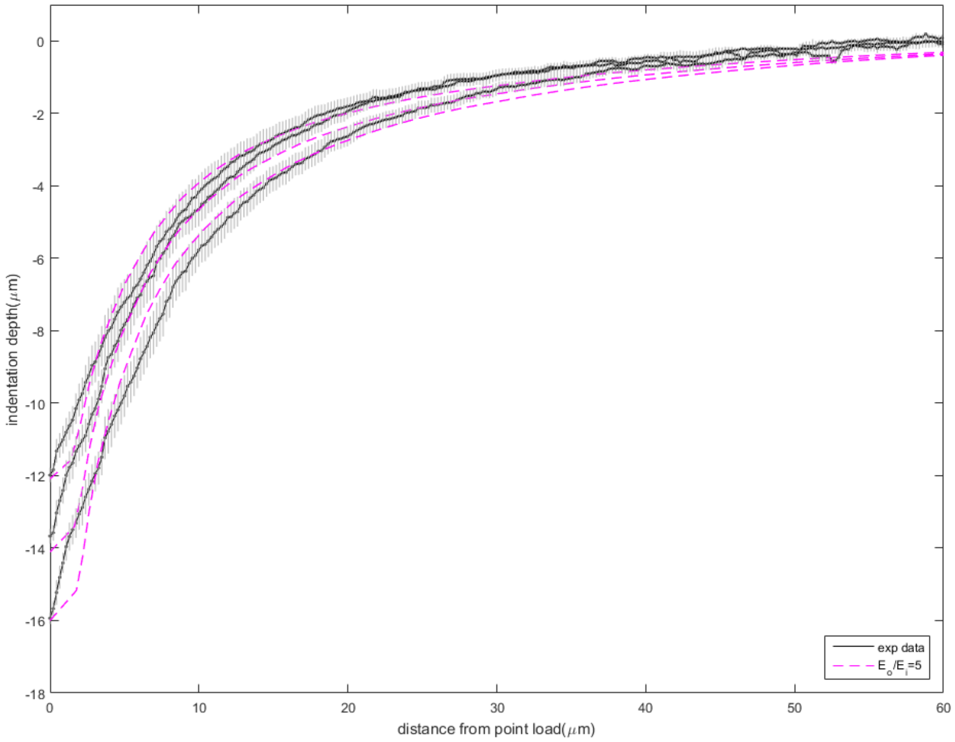

Fig. S4.
Comparison of surface deformation profiles between mean experimental data (solid lines) and numerical prediction (dashed lines) using E_o:E_i = 5 for the indentation range 12 – 16 μm.

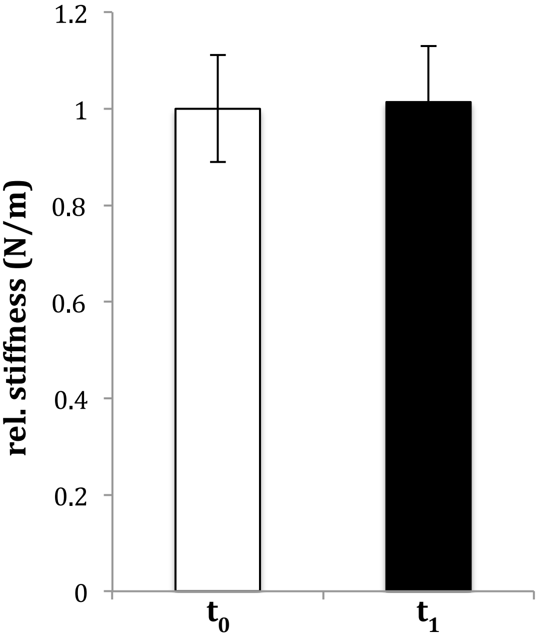

Fig S5. *C. elegans* stiffness over time. Mean stiffness values normalized to the initial measurement (t_0), where t_0 = initial measurement and t_1 = 30 min later. Adult wild-type worms indented at anterior and posterior locations in each case. Error bars show ±s.e.m for n=5 animals.

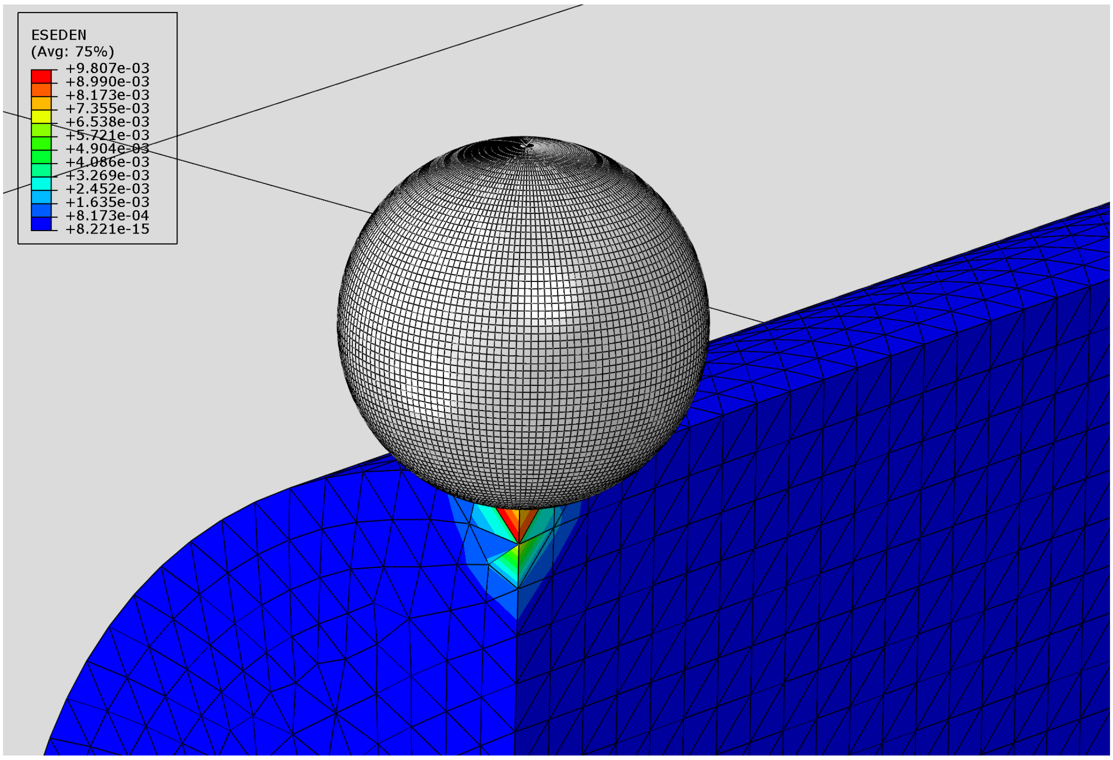

Fig. S6.
Computer simulation showing the SED (in MPa) distribution in a homogenous model, E = 140 kPa and v = 0.495, indented with a 10 μm spherical tip with a 0.49 μN indentation force.

Indentation depth μm	RMS values	R^2
2	0.119	0.923
4	0.165	0.949
6	0.164	0.980
8	0.170	0.990
10	0.253	0.986
12	0.408	0.978
14	0.468	0.977
16	0.611	0.972

Table 1.
RMS and R^2 values to qualitatively measure how well the computer simulated surface deformation profiles fit with experimental data using E_o=140 kPa, E_i=70 kPa and v=0.495
